# Cryptococcal meningitis-associated cerebrovascular injury presenting with sequential multifocal cerebral infarctions and prominent autonomic dysfunction in a non-HIV patient: a case report and focused literature review

**DOI:** 10.3389/fmed.2026.1970390

**Published:** 2026-10-14

**Authors:** Lijin Liu, Fanxin Kong, Jinfang Li, Jianjun Wang, Wei Li, Yong Jiang, Lanying Liu, Xiude Qin

**Affiliations:** 1 Department of Encephalopathy and Psychology, Shenzhen Traditional Chinese Medicine Hospital, Shenzhen, China; 2 Shanghai Mental Health Center, Shanghai Jiao Tong University School of Medicine, Shanghai, China; 3 Department of Spine Surgery, Shenzhen Traditional Chinese Medicine Hospital, Shenzhen, China

**Keywords:** autonomic dysfunction, cerebral infarction, cerebrovascular injury, cryptococcal meningitis, *Cryptococcus neoformans*, focused literature review, ischemic stroke, non-HIV

## Abstract

Cryptococcal meningitis (CM) can occur in patients without human immunodeficiency virus (HIV) infection and may be complicated by genuine cerebral infarction, creating a powerful diagnostic anchor toward primary cerebrovascular disease. We report a 66-year-old non-HIV man with type 2 diabetes mellitus and no documented major immunosuppressive condition who initially presented with dizziness and mild headache. Brain MRI showed restricted diffusion in the left brainstem, and acute ischemic stroke was diagnosed. Over the following weeks, neurological deterioration continued despite stroke-directed treatment. Repeat MRI demonstrated new infarctions involving the left cerebellar vermis, corpus callosum, and left thalamus, while the patient developed rapid cognitive decline and prominent autonomic manifestations, including orthostatic hypotension, urinary retention, constipation, vomiting, and severe sleep disturbance. Cerebrospinal fluid (CSF) examination revealed an opening pressure >300 mmH_2_O, leukocytes 128 × 10^6^/L, protein 964 mg/L, and glucose 1.28 mmol/L. India ink staining was suspiciously positive, and *Cryptococcus neoformans* was confirmed by fungal culture and metagenomic next-generation sequencing. Serial lumbar punctures and antifungal treatment with amphotericin B and fluconazole were followed by substantial clinical improvement. To contextualize the case, we conducted a focused review of published non-HIV or apparently immunocompetent reports of CM-associated cerebral infarction. The literature shows a recurring pattern of deep perforator, brainstem, cerebellar, callosal, and multifocal ischemic injury, with both small- and large-vessel mechanisms reported. However, sequential infarctions across multiple territories accompanied by a prominent progressive autonomic syndrome remain poorly documented. This case supports early CSF investigation when recurrent infarction, encephalopathic progression, and multisystem neurological features are discordant with an isolated vascular diagnosis.

## 1 Introduction

Cryptococcal meningitis (CM), caused predominantly by *Cryptococcus neoformans* and Cryptococcus gattii, is a life-threatening fungal infection of the central nervous system (CNS). Although advanced HIV infection is a major global risk factor, CM also occurs in solid-organ transplant recipients, patients receiving glucocorticoids or other immunosuppressive agents, patients with malignancy or immune dysregulation, and individuals in whom no major predisposing immune defect is identified (1–3). In non-HIV populations, the disease may be more indolent, inflammatory, or diagnostically heterogeneous, and classic fever or meningeal irritation signs may be absent.

Cerebral infarction is an important but under-recognized complication of CM. Cohort studies suggest that ischemic lesions are not exceptional in non-HIV disease. In one HIV-negative cohort, acute or subacute cerebral infarction occurred in 14 of 53 patients (26.4%) (4); in another series, ischemic stroke complicated approximately one quarter of CM cases and was typically lacunar, multiple, and acute (5). A larger neurovascular series found infarction in 13% of CM patients and linked cerebrovascular involvement to increased morbidity and mortality (6). Proposed mechanisms include basal meningeal inflammation affecting penetrating arteries, inflammatory vasculopathy, endothelial injury, thrombosis, vessel compression, and, in rare pathological reports, venous occlusion.

We report a non-HIV patient in whom true, sequential ischemic infarctions dominated the early clinical course, while rapidly progressive cognitive decline and prominent autonomic dysfunction provided important clues that the syndrome extended beyond ordinary recurrent stroke. We further performed a focused review of published non-HIV or apparently immunocompetent cases and series to define the spectrum of CM-associated cerebrovascular injury and to clarify the distinctive features of the present case.

## 2 Case presentation

### 2.1 Initial presentation and first ischemic lesion

Day 0 was defined as 10 July 2023, the date of admission to our department. On Day −29 (11 June 2023), a 66-year-old man developed persistent dizziness and mild headache. He denied fever, photophobia, and neck stiffness. His medical history was notable for type 2 diabetes mellitus. The available record documented no organ transplantation, chronic glucocorticoid exposure, other immunosuppressive treatment, or known malignancy. Cranial computed tomography at a local hospital was interpreted as showing multiple ischemic foci in the bilateral basal ganglia, and clopidogrel and atorvastatin were prescribed.

Four days later, the headache improved but dizziness worsened and neck muscle tension appeared. The dizziness was exacerbated on standing and relieved in the supine position. On Day −13 (27 June 2023), he developed marked dizziness with five episodes of vomiting and was admitted to the local hospital. During this period he also developed poor appetite, disturbed sleep, urinary retention, constipation, and an approximately 8-pound weight loss.

At the local hospital, supine blood pressure was 132/91 mmHg and immediate standing blood pressure was 104/72 mmHg. Meningeal signs were absent and HIV serology was negative. Bladder ultrasonography showed a post-void residual urine volume of 462 mL. Brain MRI demonstrated restricted diffusion in the left brainstem (Figure 1). Magnetic resonance angiography showed atherosclerotic changes in the bilateral carotid, vertebral, and basilar arteries, a fetal-type right posterior cerebral artery, and multiple moderate stenoses in the P2–P3 segment of the right posterior cerebral artery. Acute ischemic stroke was diagnosed, and antiplatelet and lipid-lowering treatment was continued.

**Figure 1:**
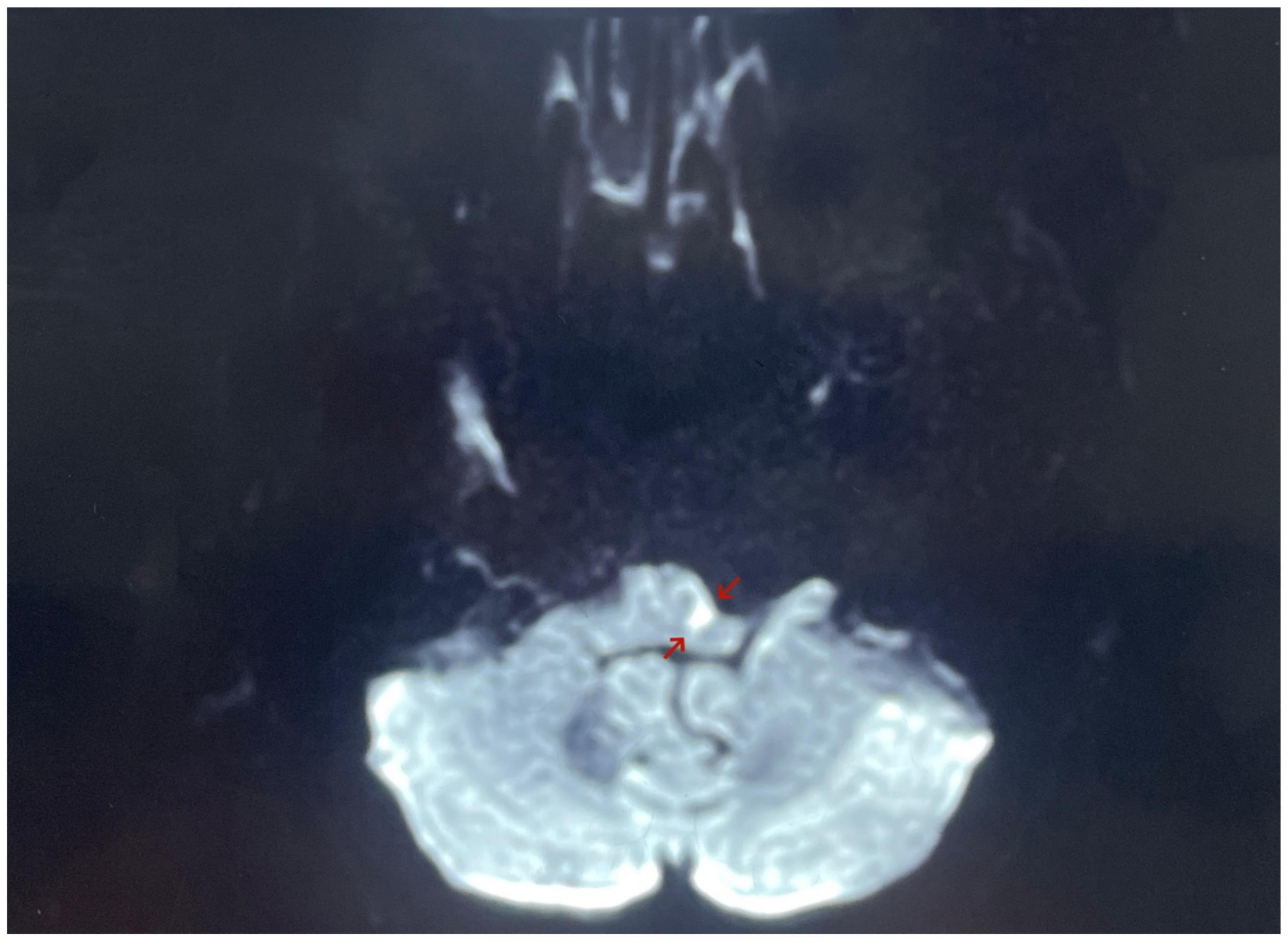
Initial brain MRI showing restricted diffusion in the left brainstem, initially interpreted as acute ischemic stroke.

Despite approximately 2 weeks of treatment, the patient deteriorated, developing lethargy, fatigue, and rapid cognitive decline. Montreal Cognitive Assessment and Mini-Mental State Examination scores were 6/30 and 12/30, respectively. He was transferred to our department on Day 0.

### 2.2 Sequential infarction and progressive multisystem neurological syndrome

At admission, the patient was oriented to self and able to follow simple commands but was intermittently disoriented to time and place. Severe orthostatic dizziness, postprandial vomiting, constipation, urinary retention, and profound sleep disturbance persisted. Supine blood pressure was 194/105 mmHg and immediate standing blood pressure was 128/92 mmHg. Neurological examination showed ptosis, with full extraocular movements, equal and reactive pupils, and no other cranial nerve abnormalities; reduced tendon reflexes in both lower limbs, and a positive Romberg test; meningeal irritation signs remained negative.

Laboratory testing showed blood neutrophils 79.2%, lymphocytes 13.6%, total WBC count 6.98 × 10^9^/L, serum sodium 128.8 mmol/L, and serum chloride 92.0 mmol/L. The glycated haemoglobin (HbA1c) level was 6.7%. Antinuclear antibody testing was positive at 1:100, whereas broader rheumatologic and humoral immune testing did not identify a major abnormality. Electromyography demonstrated sensory and motor peripheral nerve injury in the right upper and lower limbs with demyelinating and axonal features.

Review of interval brain MRI revealed new diffusion-restricted lesions involving the left cerebellar vermis, corpus callosum, and left thalamus (Figure 2). The rapid appearance of anatomically distinct lesions despite stroke-directed treatment, together with cognitive deterioration and a broader autonomic/peripheral neurological syndrome, prompted diagnostic reassessment.

**Figure 2:**
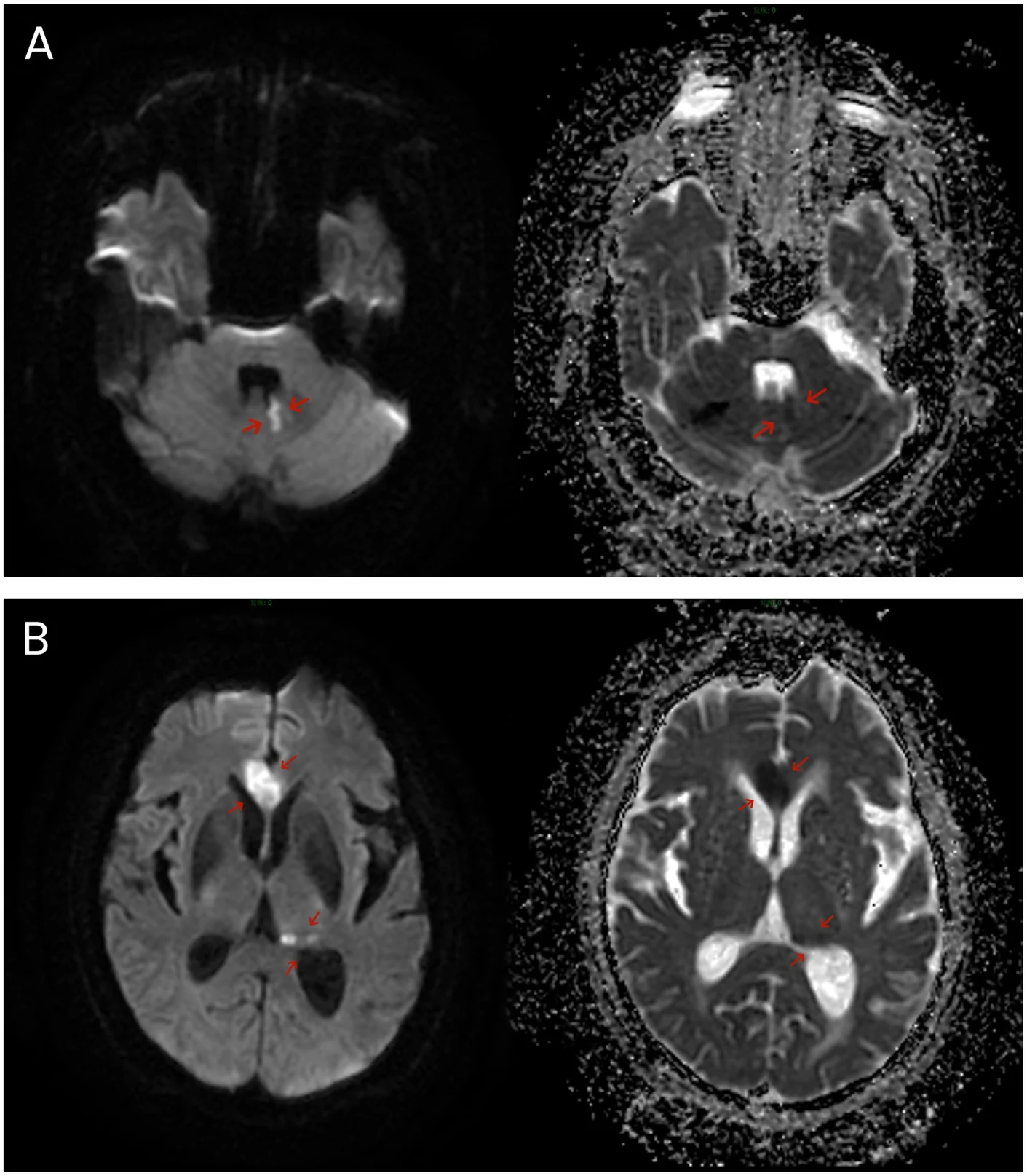
Follow-up MRI after neurological deterioration. **(A)** DWI and ADC demonstrate acute infarction in the left cerebellar vermis. **(B)** DWI and ADC demonstrate additional acute lesions involving the corpus callosum and left thalamus.

### 2.3 CSF and mycological diagnosis

Approximately 1 week after admission to our department, lumbar puncture yielded xanthochromic CSF with an opening pressure >300 mmH_2_O. CSF protein was 964 mg/L, glucose was 1.28 mmol/L, and leukocyte count was 128 × 10^6^/L, CSF leukocyte differential count showed lymphocytes 78%, monocytes 11%, and neutrophils 11%. India ink staining was suspiciously positive (Figure 3). Cryptococcal antigen titres in serum and CSF were not performed. *Cryptococcus neoformans* was subsequently confirmed by fungal culture and metagenomic next-generation sequencing, which established the diagnosis of cryptococcal meningitis. The clinical timeline is summarized in Figure 4, and the fungal culture is shown in Figure 5. Treatment consisted of serial lumbar punctures for elevated intracranial pressure and antifungal therapy with amphotericin B, flucytosine, and fluconazole. As the antifungal agents required for cryptococcal meningitis were unavailable at our institution, the patient was transferred to an external centre immediately after diagnosis for definitive management. According to follow-up information provided by the patient's family, he received continuous intravenous amphotericin B in combination with fluconazole for approximately 40 days. Although his general condition remained poor during this period, serial laboratory monitoring showed largely preserved electrolyte, hepatic, and renal function. Following discharge, oral fluconazole (16 tablets daily) was continued for approximately 1 year and was discontinued around August 2024. Dose-by-dose data and external hospital records were not available; consequently, the antifungal regimen could not be fully reconstructed, and these follow-up details are based on family recall and should be interpreted accordingly. The patient's clinical condition improved progressively after discharge. Clear functional improvement was evident by approximately 2 months, and by 6 months he was able to perform self-care and simple household activities independently. At the most recent follow-up in September 2026, his memory had improved relative to earlier assessments but remained mildly below his premorbid baseline; he was fully independent in daily living and able to go out and collect his grandson from school.

**Figure 3:**
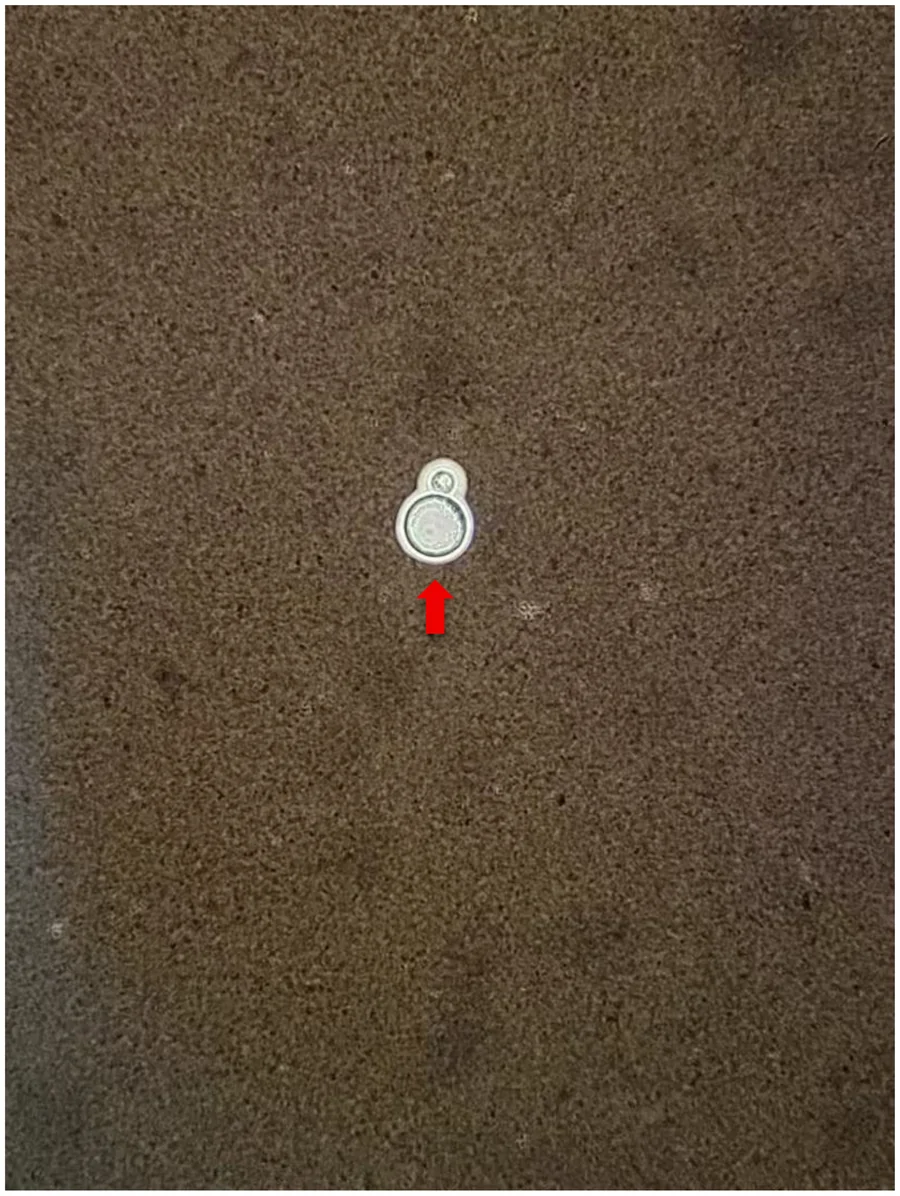
This photomicrograph illustrates an India ink mount of cerebrospinal fluid examined at ×100 magnification, revealing *Cryptococcus neoformans* with evident budding. The pale, round yeast forms are sharply contrasted against the dark brown background, permitting easy identification.

**Figure 4:**
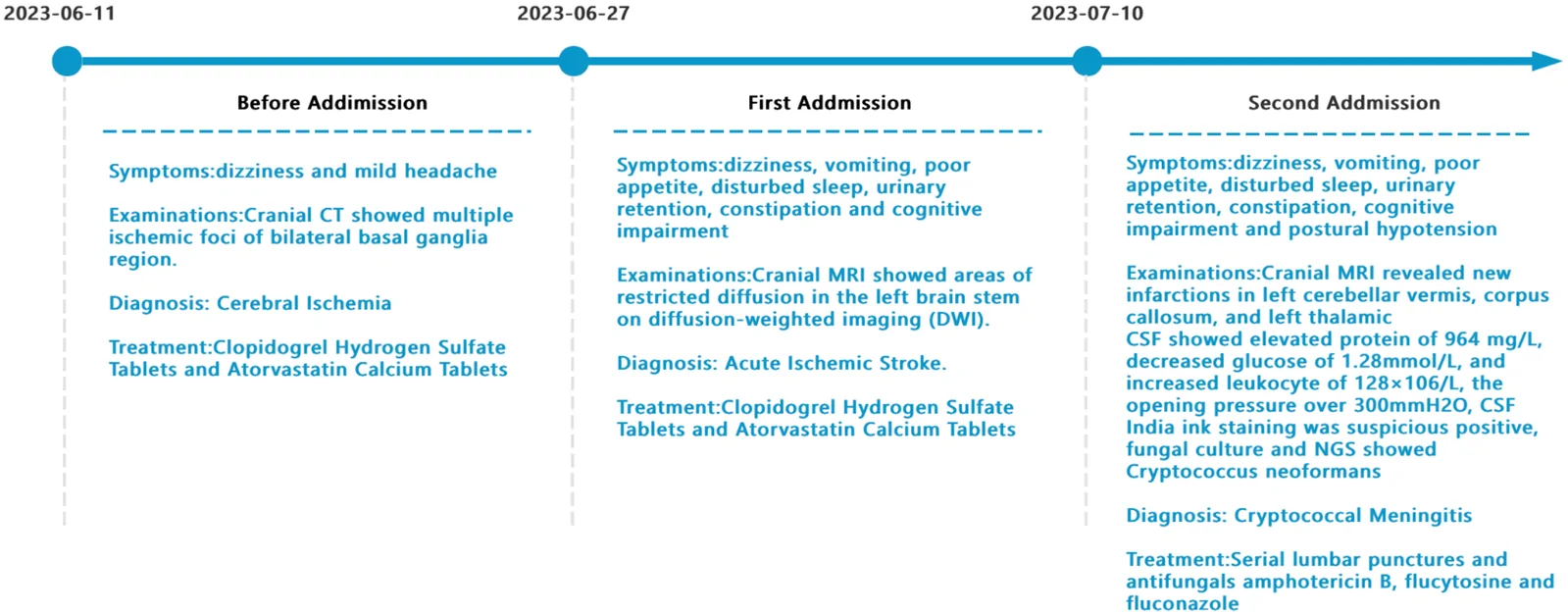
Clinical timeline showing evolution from presumed cerebral ischemia to sequential infarction, cognitive/autonomic deterioration, CSF examination, and diagnosis of cryptococcal meningitis.

**Figure 5:**
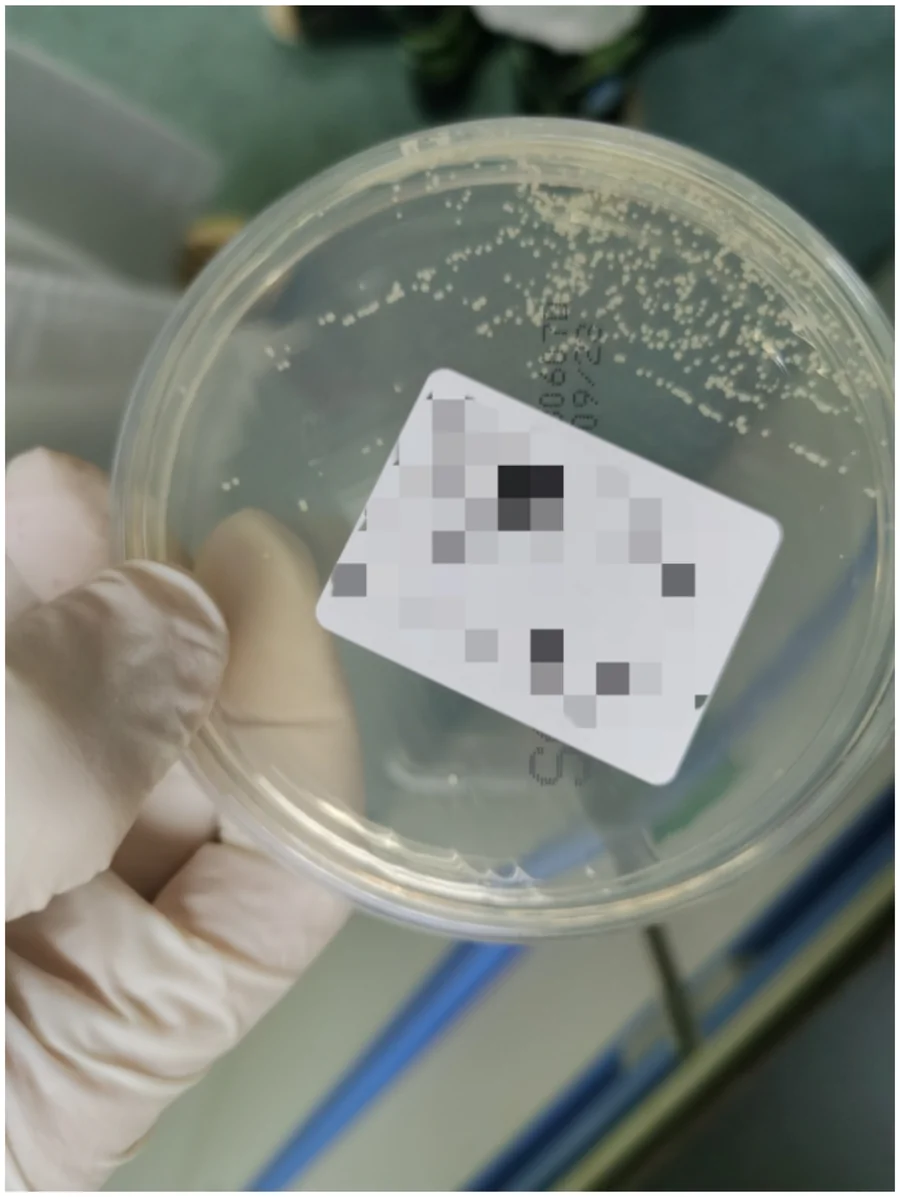
Fungal culture obtained during diagnostic evaluation and subsequently identified as *Cryptococcus neoformans*. Patient-identifying information on the plate label is obscured.

## 3 Focused literature review

### 3.1 Review strategy

We performed a focused, non-systematic literature review to contextualize the cerebrovascular phenotype of the present case. Searches were conducted in PubMed/MEDLINE and supplemented by searches of publisher-hosted full texts and reference lists using combinations of the terms “cryptococcal meningitis,” “cryptococcal meningoencephalitis,” “*Cryptococcus neoformans*,” “cerebral infarction,” “ischemic stroke,” “recurrent stroke,” “vasculitis,” “vasculopathy,” “brainstem infarction,” “cerebellar infarction,” and “corpus callosum infarction.” Priority was given to published case reports, case series, and clinical cohorts involving non-HIV patients or patients described by the original authors as immunocompetent/apparently immunocompetent. HIV-associated stroke reports were excluded from the case-level comparison table, although major mixed-host cohort studies were retained for epidemiological context. Because this was a focused review embedded in a case report rather than a systematic review, no formal PRISMA flow diagram or meta-analysis was performed.

### 3.2 Published non-HIV and apparently immunocompetent cases

The focused review identifies several recurring patterns. First, the infarctions are often genuine ischemic lesions rather than radiological mimics. Second, deep perforator and posterior-circulation structures recur across reports, including the midbrain, basal ganglia, thalamus, cerebellum, and corpus callosum (7–12). Third, multifocal or recurrent infarction has been repeatedly described, including cases with involvement of multiple vascular territories and, more recently, large-vessel vasculitic infarction (10, 11, 13, 14). Finally, although autonomic disturbances may occur in cryptococcal CNS disease, a syndrome dominated by orthostatic hypotension, urinary retention, constipation, vomiting, and sleep disturbance accompanying sequential multifocal infarction is not a prominent feature of the published non-HIV cerebrovascular case literature reviewed here (Table 1).

**Table 1:** Published non-HIV and apparently immunocompetent cases.

Study	Age/sex	Host status	Initial/core presentation	Infarct/vascular pattern	Autonomic/other features	Microbiological/vascular evidence	Relevance
Kalita et al., 1999 (7)	20/M	Non-HIV	Headache, fever 1 month → sudden left hemiplegia, left arm tremor, titubation → deep coma	Right midbrain crus infarct	Resting tremor, titubation, quadriparesis	CSF: protein 66, glucose 70, 10 lymphs; India ink negative; CrAg positive	First report of brainstem infarct as presenting phenotype
Lai et al., 2017 (8)	60/F	Immunocompetent; HIV negative	Dizziness, dysarthria, gait deviation → headache, fever, meningeal sign	Left midbrain lacunar infarct; caudate and callosal edema; MRA normal	Mild right hemiparesis; no dysautonomia	CSF: OP 25 cmH₂O, WBC 156, glucose 15, protein 144; culture positive; CrAg 1:4	Brainstem infarct in non-HIV host with early fever/meningeal clues
Zhou et al., 2018 (9)	81/F	No predisposing factor	Headache without fever → weakness, psychosis, lethargy; initially misdiagnosed as infarct	Multiple ischemic foci; severe arterial stenosis on MRA	Later hydrocephalus and neuropsychiatric decline	India ink positive; LP delayed at first admission	Diagnostic anchoring on stroke in elderly immunocompetent patient
Sengel et al., 2021 (10)	59/M	Immunocompetent; HIV negative	Headache, slow speech, inability to walk, weakness	Multiple cerebral-cerebellar lesions; caudate and internal capsule infarcts; hydrocephalus	Confusion and aphasia during relapse	India ink negative initially; Biofire panel positive; culture positive	Multiple infarcts in immunocompetent host; value of multiplex PCR
Tarhan et al., 2021, Case 2 (11)	35/M	No immunodeficiency; hyperlipidemia; seizure disorder	Breakthrough seizure; punctate infarcts found incidentally; later diplopia, aphasia, headache, weakness	New cerebellar and occipital infarcts; splenial infarct; meningeal enhancement; spinal lesions	NIHSS 8; facial palsy, ataxia	CSF: OP 24, WBC 150 K (61% lymphs), glucose 15; CrAg positive	Multifocal infarcts, callosal and spinal involvement without known immunodeficiency
Ansari & Rahimzadeh, 2024 (12)	33/F	Immunocompetent; HIV/HBV/HCV negative	Severe headache, mild fever 7 days; no focal deficit initially	Splenium of corpus callosum infarct; follow-up internal capsule hyperintensity	Later decreased consciousness; intubated	CSF: OP 25, glucose 39, protein 189, WBC 28 (98% lymphs); India ink negative; CSF CrAg DNA positive	Corpus callosum infarct in immunocompetent host
Sahib et al., 2025 (13)	40/F	Immunocompetent; HIV negative; CD4 513	Recurrent deficits over 3 months: headache → right hemiparesis → hemianopia, seizures	Multiple territory infarcts; DSA: severe narrowing of ICA, ACA, MCA	Non-fluent aphasia; hemianopia; hemiparesis; mRS 5	CSF: OP 25, 100 cells (90% lymphs), glucose 49; India ink negative; culture sterile; CrAg positive	Recurrent large-vessel vasculitic infarcts in truly immunocompetent host
Ye et al., 2025 (14)	Early 60s/M	Non-AIDS; HTN and T2DM (poorly controlled)	Recurrent left limb weakness over 4 months; later headache, stiff neck, fever, coma	Bilateral cerebellar infarcts; later brainstem and hemispheric infarcts; meningeal enhancement	Slurred speech; hemiparesis; later coma, respiratory and renal failure; died	CSF: India ink positive; culture positive; blood CrAg positive	Recurrent stroke as first manifestation; fatal outcome despite antifungal therapy
Present case	66/M	HIV negative; T2DM	Dizziness, mild headache → brainstem infarct → cognitive/autonomic decline	Sequential brainstem, cerebellar vermis, callosal, and thalamic infarcts	Orthostatic hypotension, urinary retention, constipation, vomiting, sleep disturbance; neuropathy	CSF OP >300; culture and mNGS positive for C. neoformans	Sequential multifocal infarcts plus prominent autonomic syndrme without fever or meningeal signs

### 3.3 Cohort context in non-HIV cryptococcal meningitis

Case reports alone can exaggerate rarity and novelty, so we also considered cohort-level evidence. A 2016 case-control study of HIV-negative CM identified 10 patients with acute/subacute cerebral infarction; all infarcts were lacunar, four patients had multiple infarctions, and hydrocephalus and smoking were independently associated with infarction (18). Mishra et al. subsequently reported neurovascular infarction in 13% of a larger CM cohort; all infarcts were lacunar, half of acute infarcts were multiple, and basal ganglia involvement predominated (6). Vela-Duarte et al. found ischemic stroke in approximately 26% of CM cases, with strokes typically acute, lacunar, and multiple (5). More recently, Tu et al. reported acute/subacute infarction in 14 of 53 HIV-negative CM patients (26.4%), with frequent basal ganglia–corona radiata and brainstem–cerebellar involvement and an association with poorer outcomes (4). These data support the concept that ischemic injury is a clinically meaningful complication of non-HIV CM rather than an isolated curiosity.

## 4 Discussion

### 4.1 The key diagnostic error: treating the infarct as the disease rather than a complication

The first major lesson from this case is that CM-associated cerebrovascular injury can generate a convincing diagnosis of acute ischemic stroke because the infarcts are real. The initial brainstem DWI lesion and coexisting atherosclerotic changes on MRA made a primary vascular explanation plausible. The diagnostic problem arose when that explanation was not revisited despite continued deterioration. Three red flags accumulated: new infarctions appeared in anatomically distinct territories over a short interval; rapid cognitive decline was disproportionate to the initial brainstem lesion; and a multisystem autonomic/peripheral neurological syndrome evolved in parallel.

The focused review reinforces this interpretation. Brainstem infarction has been reported as a presenting manifestation of CM (7, 8), multiple infarctions in apparently immunocompetent hosts have been documented (10, 11), callosal infarction has been reported (12), and recurrent acute stroke has now been described in recent non-AIDS CM reports (13, 14). Therefore, novelty should not be claimed for any one lesion location. The distinctive contribution of the present case lies in the sequential combination of brainstem, cerebellar vermian, thalamic, and callosal infarctions together with prominent autonomic dysfunction and rapid cognitive decline in the absence of classic fever or meningeal signs during the key diagnostic period.

### 4.2 Cerebrovascular mechanisms: beyond a single ‘cryptococcal vasculitis’ label

The mechanisms of ischemic injury in CM are probably heterogeneous. Basal meningeal inflammation can affect small penetrating arteries and arterioles; inflammatory exudates may narrow or compress vessels; endothelial injury and thrombosis may contribute; and larger-vessel vasculopathy or arteritis has been reported in selected cases (6, 11, 13). Pathological literature also raises the possibility of inflammatory venous occlusion in unusual infarct patterns. This heterogeneity is important because the term ‘cryptococcal vasculitis’ is often used too broadly in individual case reports.

In the present patient, no vessel-wall MRI, catheter angiography, or histopathological examination was performed. We therefore deliberately use the broader term ‘CM-associated cerebrovascular injury’ rather than asserting proven cryptococcal vasculitis. Coexisting intracranial atherosclerosis also provides a competing vascular substrate. Nonetheless, the rapid emergence of infarctions across several territories during untreated CNS infection, followed by microbiological confirmation of C. neoformans, strongly supports an infection-associated contribution.

### 4.3 Autonomic dysfunction as a diagnostic clue, not a proven single mechanism

The autonomic syndrome is the second feature that distinguishes the present case from most published non-HIV CM-stroke reports. Orthostatic hypotension was documented twice, urinary retention was supported by a post-void residual volume of 462 mL, and constipation, vomiting, and severe sleep disturbance evolved with the neurological decline. These findings broadened the phenotype beyond an isolated focal stroke.

However, causality should be interpreted conservatively. The autonomic abnormalities may have been multifactorial. First, spinal arachnoiditis is a plausible anatomical mechanism: chronic meningeal inflammation can extend to the spinal subarachnoid space, causing nerve root adhesion and compression of sacral parasympathetic fibers, as described by Panackal et al. in non-HIV cryptococcal meningoencephalitis (15). In the present case, spinal MRI was not performed, so this remains inferential. Second, a post-infectious inflammatory response syndrome (PIIRS) may contribute (16), since persistent cytokine-mediated injury can involve brainstem autonomic centers or peripheral autonomic ganglia even after microbiological clearance. Third, intracranial hypertension and hyponatremia may impair brainstem perfusion and hypothalamic–pituitary regulation (17). Multifocal CNS lesions, electrophysiologically confirmed peripheral neuropathy, and diabetic autonomic neuropathy may further compound the dysfunction.

Accordingly, we do not attribute every autonomic feature directly to Cryptococcus. Their importance is diagnostic: the cluster signaled that the illness could not be adequately unified by the initial ischemic lesion alone.

### 4.4 Why CSF evaluation should occur earlier in selected ’stroke’ patients

The absence of fever and meningeal irritation signs can create false reassurance in non-HIV CM. In the present patient, the decisive diagnostic shift occurred only after lumbar puncture demonstrated markedly elevated intracranial pressure, pleocytosis, hyperproteinorrachia, and hypoglycorrhachia. Fungal culture then provided conventional microbiological confirmation, while metagenomic next-generation sequencing served as concordant evidence rather than the sole basis for diagnosis.

The practical implication is not that lumbar puncture should be performed routinely in acute ischemic stroke. Rather, CSF analysis should be considered when the clinical trajectory is discordant with the vascular diagnosis—particularly when infarcts are recurrent or sequential, occur in multiple territories without a satisfactory embolic explanation, or are accompanied by progressive encephalopathy, cranial neuropathies, unusual meningeal or hydrocephalic imaging, unexplained systemic/autonomic features, or inflammatory clues.

### 4.5 Treatment and outcome

Modern management of CNS cryptococcosis requires effective fungicidal induction therapy together with active management of elevated intracranial pressure (19). In non-HIV patients, standard regimens consist of three phases: induction with amphotericin B plus flucytosine (or fluconazole when flucytosine is unavailable) for at least 4 weeks, consolidation with fluconazole 400–800 mg/day for at least 8 weeks, and maintenance with fluconazole 200 mg/day for 6–12 months (19). In this case, serial lumbar punctures and amphotericin B- and fluconazole-based therapy were followed by meaningful functional recovery over 6 months. Because complete dose, formulation, serial opening-pressure, and culture-conversion data were not available in the source record, we avoid reconstructing undocumented treatment details.

### 4.6 Limitations of the case and focused review

This report has several limitations. First, although the patient was HIV negative and lacked documented major immunosuppression, diabetes mellitus was present and detailed cellular immune phenotyping was unavailable; ‘immunocompetent’ would therefore be overly categorical. Second, the mechanism of the infarctions remains inferential because vessel-wall imaging and pathology were not performed. Third, complete antifungal dosing and serial microbiological follow-up were unavailable. Fourth, the literature review was intentionally focused rather than systematic; it prioritized clinically comparable non-HIV reports and may not capture every historical publication, non-English report, or conference abstract. For these reasons, the review is used to contextualize phenotype rather than to estimate prevalence.

## 5 Conclusions

Cryptococcal meningitis in non-HIV patients can produce true cerebral infarctions and may present as recurrent or sequential cerebrovascular disease. The published literature demonstrates a broad ischemic spectrum involving deep perforator territories, brainstem, cerebellum, corpus callosum, and occasionally larger vessels. In the present case, the combination of sequential multifocal infarction, rapid cognitive deterioration, and prominent autonomic dysfunction was the critical clue that the syndrome extended beyond ordinary stroke. When the evolution of recurrent ischemic lesions is clinically discordant with a conventional vascular mechanism, early CSF examination and mycological testing should be considered even in the absence of fever or meningeal signs.

## Data Availability

The original contributions presented in the study are included in the article/Supplementary Material, further inquiries can be directed to the corresponding authors.
